# Differential profiles of soluble and cellular toll like receptor (TLR)-2 and 4 in chronic periodontitis

**DOI:** 10.1371/journal.pone.0200231

**Published:** 2018-12-20

**Authors:** Hawra AlQallaf, Yusuke Hamada, Steven Blanchard, Daniel Shin, Richard Gregory, Mythily Srinivasan

**Affiliations:** 1 Department of Periodontics and Allied Dental Programs, School of Dentistry, Indiana University–Purdue University Indianapolis, Indianapolis, Indiana, United States of America; 2 Department of Biomedical and Applied Sciences, School of Dentistry, Indiana University–Purdue University Indianapolis, Indianapolis, Indiana, United States of America; 3 Department of Oral Pathology, Medicine and Radiology, School of Dentistry, Indiana University–Purdue University Indianapolis, Indianapolis, Indiana, United States of America; Medical University of South Carolina, UNITED STATES

## Abstract

Chronic periodontitis is a common inflammatory disease initiated by a complex microbial biofilm and mediated by the host response causing destruction of the supporting tissues of the teeth. Host recognition of pathogens is mediated by toll-like receptors (TLRs) that bind conserved molecular patterns shared by large groups of microorganisms. The oral epithelial cells respond to most periodontopathic bacteria via TLR-2 and TLR-4. In addition to the membrane-associated receptors, soluble forms of TLR-2 (sTLR-2) and TLR-4 (sTLR-4) have been identified and are thought to play a regulatory role by binding microbial ligands. sTLR-2 has been shown to arise from ectodomain shedding of the extracellular domain of the membrane receptor and sTLR-4 is thought to be an alternate spliced form. Many studies have previously reported the presence of elevated numbers of viable exfoliated epithelial cells in the saliva of patients with chronic periodontitis. The objective of this study was to investigate the potential value of salivary sTLR-2 and sTLR-4 together with the paired epithelial cell-associated TLR-2/4 mRNA as diagnostic markers for chronic periodontitis. Unstimulated whole saliva was collected after obtaining informed consent from 40 individuals with either periodontitis or gingivitis. The sTLR-2 and sTLR4 in saliva was measured by enzyme-linked immunosorbent assay. The TLR-2 and TLR-4 transcript in the epithelial cells in saliva was measured by real time polymerase chain reaction. While levels of sTLR-2 exhibited an inverse correlation, sTLR-4 positively correlated with clinical parameters in the gingivitis cohort. Interestingly, both correlations were lost in the periodontitis cohort indicating a dysregulated host response. On the other hand, while the sTLR-2 and the paired epithelial cell associated TLR-2 mRNA exhibited a direct correlation (r^2^ = 0.62), that of sTLR4 and TLR-4 mRNA exhibited an inverse correlation (r^2^ = 0.53) in the periodontitis cohort. Collectively, assessments of salivary sTLR2 and sTLR4 together with the respective transcripts in the epithelial cells could provide clinically relevant markers of disease progression from gingivitis to periodontitis.

## Introduction

Periodontitis is a biofilm-induced, host-immune mediated, chronic inflammatory disease that if undiagnosed and untreated leads to the destruction of the supporting apparatus of the teeth [1, 2]. Nearly 46% of US adults, ages 30 years and older, representing 64.7 million individuals, suffer from periodontitis, with 8.9% or 12.3 million having severe periodontitis [3]. Changing composition of the dental plaque biofilm from commensal to pathogenic flora promotes clinical progression of the disease from a periodontally healthy status to destructive periodontitis [2]. Pathologically, the progression involves skewing of the host response from predominantly protective innate immune responses to an exaggerated response characterized by increased pro-inflammatory cytokines, eicosanoids, reactive oxygen species and matrix metalloproteinases that mediate destruction of the tooth supporting tissues [4, 5].

The primary objectives of periodontal therapy are removal of pathogenic biofilm and debridement of the affected tissues in order to facilitate resolution of inflammation and healing of tissue attachment around teeth. Together with meticulous personal oral hygiene, this should reduce bacterial accumulation [6, 7]. However, very often in individuals with chronic periodontitis (CP) maintenance of periodontal health requires periodic assessments and intervention [8, 9]. Current methods of diagnosing and monitoring periodontitis include measurement of probing depths, recessions, clinical attachment levels (CAL), bleeding on probing (BOP), presence of plaque, suppuration and radiographic bone loss [1]. While these methods are excellent in detecting tissue destruction and established disease, they are less efficient in identifying active disease at the time of diagnosis or predict future risk [1, 10]. Hence, over the years there has been active search for identifying biomarkers as supplemental diagnostic and risk assessment tools for managing periodontitis [11].

Host recognition of microorganisms is primarily mediated by pattern recognition receptors (PRRs), such as toll like receptors (TLR) that act by binding conserved molecular features, termed pathogen-associated molecular patterns (PAMPs) that are shared by large groups of microorganisms. To date thirteen mammalian TLRs and many of their ligands have been identified [12, 13]. In addition to the membrane-associated TLRs, soluble forms of TLRs (sTRLs) have been identified in serum, urine, tears and saliva [14–16]. The sTLRs are thought to function as negative regulators and inhibit membrane associated TLR mediated signaling pathways [17]. Based on their ligand preferences, TLR-2 and TLR-4 respond to most periodontal pathogens by binding the peptidoglycan of the gram positive and the lipopolysaccharide of the gram negative bacterial cell walls, respectively [18, 19]. Both, TLR-2 and TLR-4 work with the co-receptor CD14 in binding periodontal pathogens. Altered expression profiles of CD14, TLR-2 and TLR-4 have been previously reported in periodontitis [13, 20, 21]. Additionally, periodontal pathogens have been shown to induce TLR-2 or TLR-4 mediated signaling and up-regulate cytokine production in oral epithelial cells [22, 23].

Early investigations on biomarkers for periodontitis focused on the assessment of serum and gingival crevicular fluid for inflammatory molecules [24]. However, in the recent past considerable efforts have been made in identifying markers in saliva, an easily accessible biospecimen that is amenable for painless and frequent collection [25, 26]. Different classes of molecules such as cytokines, matrix metalloproteinases, and micro RNAs in saliva have been evaluated as biomarkers for periodontitis [27–30]. Recently, levels of microbial ligands for TLR-2 and TLR-4 have been reported to be higher in the saliva of periodontitis patients [31]. Interestingly, while the sTLR-2 has been shown to exhibit a decreasing trend in saliva, that of sTLR-4 present an increasing trend in periodontitis [7, 32, 33].

In addition to the solutes in clarified saliva, previously others and we have evaluated the characteristics of the cells in saliva in multiple clinical conditions [26, 34, 35]. In a recent detailed analysis of cells in saliva, Theda et al., reported that the non-keratinized surface cells constitute the most abundant epithelial cells in saliva [35]. In CP, the number of exfoliated epithelial cells are higher resulting from the increased exfoliation secondary to inflammation [36–38]. Interestingly, we observed that much like the primary human gingival epithelial cells, stimulation of epithelial cells in periodontitis saliva with TLR-2 or TLR-4 specific ligands induced cytokine secretion with differential kinetics and up-regulated TLR-2 and TLR-4 mRNAs [23, 39]. The purpose of this study was to detect potential correlations between the soluble and epithelial cell associated expression of TLR-2 and TLR-4 in CP and possible use as biomarkers to assess the status and progression of the periodontal diseases.

## Materials and methods

### Study population and clinical measurements

The study included 20 individuals exhibiting clinical features of generalized moderate to severe CP [> 30% of sites having >4 mm of clinical attachment loss (CAL)], 20 age-matched individuals with gingivitis [no CAL site >2mm, sites with CAL between 1-2mm not >15%, no radiographic evidence of bone loss] and 10 individuals with normal oral health [minimal to no (0-1mm) CAL] [7]. Periodontal diagnoses were validated with radiographic evidence of bone loss with the help of recent full-mouth radiographs [40]. The study was approved by the Institutional Review Board at Indiana University Purdue University at Indianapolis (IUPUI).

### Saliva collection and isolation of epithelial cells

Unstimulated whole saliva (UWS) was collected on the second visit following clinical diagnosis of CP [7, 16]. The subjects refrained from eating or drinking for 1 h prior to saliva collection. Subjects were seated, asked to swallow and UWS was collected with their head tilted towards one side by passive drooling for 10 min into a 15 ml chilled centrifuge tube. The samples were transported on ice to the laboratory for processing and immediately processed. All UWS samples were centrifuged at 250 x g for 10 min at 4°C. Cellular sediment was re-suspended in isotonic saline at 1:10 volumetric ratio and two drops of Zap-O-globin to lyse blood corpuscles and then centrifuged at 1,271.7 x g for 10 min at 4°C [34, 39]. The cell suspension was then filtered through a membrane of 20-micron pore size. The membrane-trapped salivary epithelial cells (SEC) enriched preparation was assessed by light microscopy for appropriate morphology, reconstituted in RPMI-1640 (Mediatech Inc., Minnesota, MN, USA), supplemented with 5% fetal bovine serum (Hyclone fetal bovine serum, Thermo Scientific, Logan, UT, USA) and 5% dimethylsulfoxide (Sigma-Aldrich, St Louis, MO, USA), and stored at -80°C until further analysis.

### ELISA for cytokine and sTLRs

Protein content of clarified saliva was determined by spectrophotometry using the Bradford method to increase the sensitivity of detection of low abundant proteins. Each UWS sample was depleted of amylase and immunoglobulins by incubating serially with anti-human amylase mAb (1:2500; Abcam, Cambridge, MA, USA) and protein G beads (Miltenyi Biotec Inc Auburn, CA) at 4°C. One microgram of protein from each treated UWS sample was assessed for the presence of IL-8, sCD14, sTLR-2/sTLR-4 by ELISA [7, 16, 41]. sTLR-2 and sTLR-4 were detected in duplicate using anti-human TLR-2 mAb and anti-human TLR-4 mAb (R&D Systems, MN), respectively. Bound antibodies were detected using HRP-conjugated secondary antibody followed by TMB (3,3′,5,5′-tetramethylbenzidine) substrate (Pharmingen, San Diego, CA, USA). Purified recombinant human TLR-2Fc and TLR-4Fc (R&D Systems) was used to develop a standard curve. The concentration sCD14 was measured by sandwich ELISA using the commercially available kit (R&D Systems) and that of IL-8 was determined using the OPT EIA kit (BD Biosciences, CA). Absorbance at 450 nm was measured in a microplate reader (Biorad Laboratories, Hercules, CA, USA).

### Quantitative real-time PCR for epithelial TLR-2 and TLR-4

Total cellular RNA was isolated from SEC using a Qiagen RNA isolation kit (Invitrogen) and reverse-transcribed using an iScript cDNA synthesis kit (Biorad, Austin, TX, USA). The concentration of the cDNA was measured at 260 and 280 nm by the Gensys5model UV-visible spectrophotometer (ThermoelectronicCorp.,CA). Real-time PCR was performed by using the SYBR green/ROX qPCRmaster mix (SABiosciences, Frederick, MD) according to manufacturer's recommendations on the CFX96 Touch Real-Time PCR Detection System (Biorad laboratories, Hercules, California, USA). Each reaction contains 2ng/ml of cDNA, 2×12.5 μl of SYBR green Master Mix and 1 μl of 10 μM of primers to a total volume of 25 μl. Message for small proline-rich protein 2a (SPRR2a) abundantly expressed in differentiated squamous epithelium and that of the housekeeping gene [42, 43], glyceraldehyde-3-phosphate dehydrogenase (GAPDH), were amplified as control [39]. Primers used were as follows: GAPDH-F:5’CATGACCACAG TCCATGCCATCACT-3’, GAPDH-R:5’ATGACCTTGCCCACAGCCTT-3’; TLR-2F:5’ACCTGTGTGA CTCTCCATCC-3, TLR-2R:5’GCAGCATCATTGTTCTCTC-3’; TLR-4F:5’TTCCTCTCCTGCGTGAG AC-3’, TLR-4R:5’TTCATAGGGTTCAGGGAC AG-3’ and SPRR2aF:5’AGTGCCAGCAGAAATATCC TCC-3’, SPRR2a-R:5’GAACGAGGTGAGCCAA ATATCC -3’. TLR-2 and TLR-4 mRNA in macrophages were amplified as positive controls. The PCR products were visualized and images acquired. The magnitude of change in the TLR-2 and TLR-4 mRNA with respect to that of SPRR2a mRNA was expressed as 2^-ΔCt^.

### Statistical methods

Statistical differences in the expression of TLRs between the healthy, gingivitis and CP cohorts were determined using two-sample t-tests. The distribution of the expression levels was examined and a transformation of the data (e.g., natural logarithm) or nonparametric tests was used. Plots and Pearson correlation coefficients were used to test the association between soluble and cellular TLRs expression. A 5% significance level was used for all tests.

## Results

### Demographic and clinical features

The control cohort included two groups the normal oral health group that exhibited no overt signs of gingival inflammation, no CAL and minimal BOP (<5%), and the gingivitis group with minimal CAL (1-2mm), no overt clinical signs of gingival inflammation, and minimal bleeding on probing (BOP) scores. The CP group included individuals exhibiting severe clinical inflammation, high BOP scores, CAL > 4mm in 30% of sites and radiographic evidence of bone loss. A modified Schei ruler was used to measure bone loss, and an average measurement of the mesial and distal bone levels was obtained [40]. The Ramfjord teeth were selected for measurement, and if a tooth was not present, the tooth distal to it was utilized. Twelve radiographs were randomly selected, and an inter/intra examiner measurement calibration was performed (HA and YH). An interclass correlation coefficient (ICC) > 0.9 was achieved prior to the actual measurements. Patients with a diagnosis of aggressive periodontitis, any known systemic illness, history of routine use of antibiotics/anti-inflammatory therapy within the past 6 months, subjects with oral mucosal lesions and present or past history of smoking were excluded from both groups.

The average age of the normal cohort was 44.3+/-10.2, that of the gingivitis cohort was 46.10 ± 11.55 years and the CP group was 51.25 ± 13.77 years. The male to female ratio was 4:6 in the normal, 5:11 in the gingivitis and 15:3 in the periodontitis cohorts. Periodontal measurements, including plaque index, bleeding on probing and percentage of sites with pocket depth of >4 mm and mean clinical attachment levels of >4 mm were significantly higher in the periodontitis cohort. In the CP group more than 50% of sites exhibited clinical attachment loss of >4 mm, and a 20.83 ± 6.41 percentage of bone loss, meaning that this cohort is a good representation of moderate CP (Table 1).

**Table 1:** Demographic and clinical features.

Patient Profile	Normal oral health	Gingivitis N = 20 (Mean ± SD)	Periodontitis N = 20 (Mean ± SD)
*Age (years)*	29.2±1.9	46.10 ± 11.55	51.25 ±13.77
*Gender ratio (M/F)*	3:7	5:11	15:5
*PD (mm)*	0.9±0.3	2.17 ± 0.321	3.55 ± 0.88*^,^^*#*^
*CAL (mm)*	1.1±0.3	2.22 ± 0.31	3.97 ± 1.01*^,^^*#*^
*BOP (%)*	1.9±0.06	4.77 ± 7.14	38.1 ± 24.15*^,^^*#*^
*PI (%)*	13± 1.3	19.88 ± 30.7	63.28 ± 22.49*^,^^*#*^
*Sites of PD > 4 mm (%)*	1.5±0.4	2.11 ± 3.78	39.33 ± 22.47*^,^^*#*^
*Sites of CAL > 4mm (%)*	1.7±0.5	2.90 ± 3.92	51.98 ± 18.67*^,^^*#*^
*Average Bone loss (%)*	0.332±0.04	0.530 ± 0.81	20.83 ± 6.41*^,^^*#*^

PD: Periodontal depths, CAL: clinical attachment loss, BOP: Bleeding on probing, PI: plaque index.

* represents p<0.05 as compared with the gingivitis cohort and

# is p<0.05 as compared with the normal oral health cohort.

Biomarker assessment in clarified saliva.

**Fig 1:**
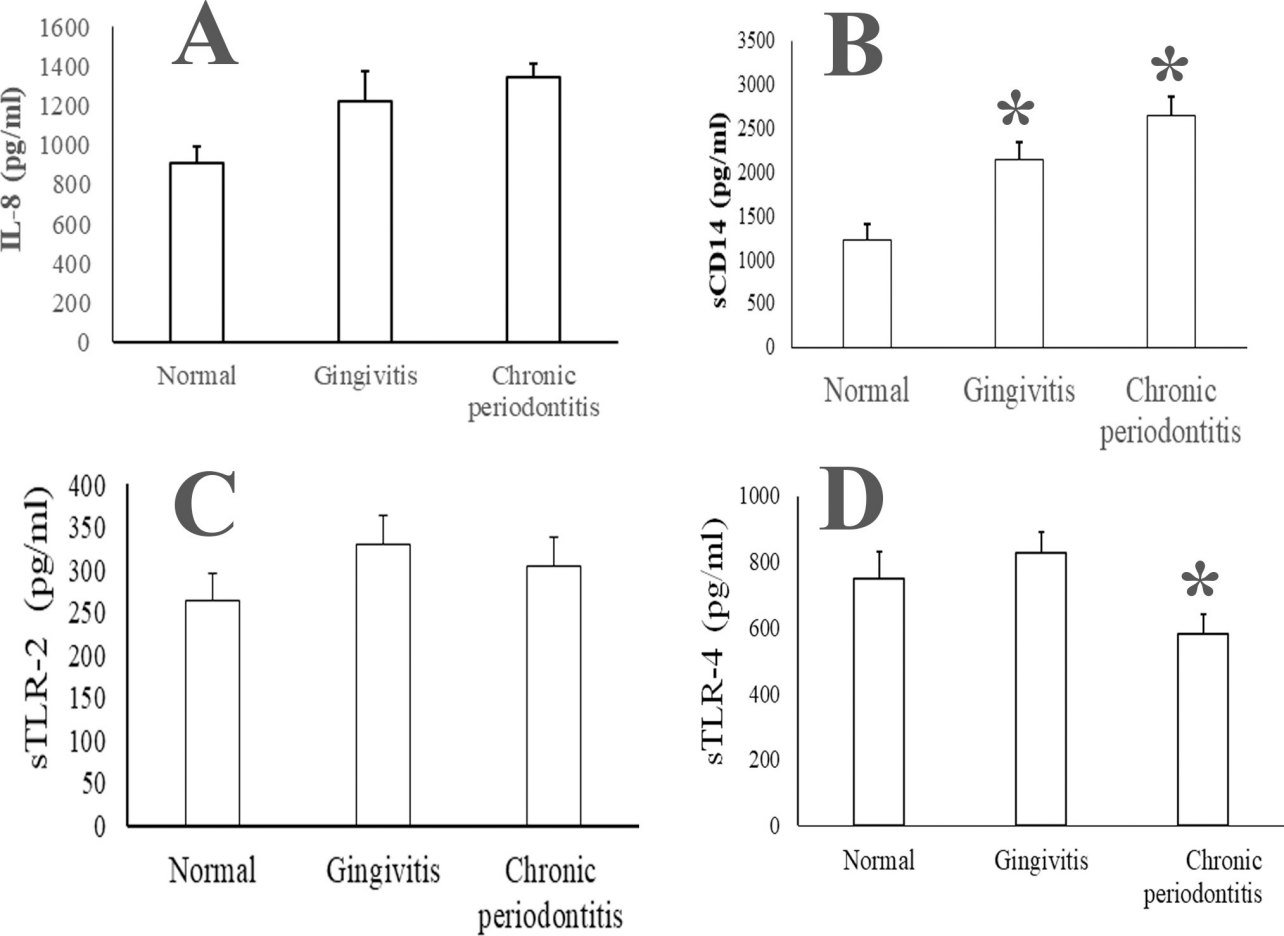
Salivary IL-8, sCD14, sTLR-2 and sTLR-4 in chronic periodontitis. Unstimulated whole saliva (UWS) was collected from 10 orally healthy individuals, 20 patients with gingivitis and 20 patients with chronic periodontitis. (A) UWS IL-8 (A) is marginally higher and that of sCD14 (B) is significantly higher in chronic periodontitis as compared to that in normal UWS samples. The concentration of both sTLR-2 (C) and sTLR-4 (D) is lower in chronic periodontitis.

Several studies have evaluated multiple cytokines as potential biomarkers for CP. Discrepancies in observations have been largely attributed to the difficulty in differentiating active vs stable disease at the time of diagnosis [11, 25, 27]. We observed that the mean IL-8 concentration in the clarified saliva was significantly higher in the CP cohort as compared to that in the normal oral health group. However, no significant difference was observed in the IL-8 concentration between the normal and gingivitis or the gingivitis and the CP groups (Fig 1A). We also assessed the concentration of sCD14, the coreceptor for both TLR-2 and TLR-4 in clarified saliva. The sCD14 was significantly higher in both the gingivitis and the CP groups as compared to that in the normal oral health cohort (Fig 1B). Previously, others and we have reported similar observations of elevated sCD14 in clarified saliva in CP [7, 44, 45]. PAMPs of TLR2 and TLR4 have been shown to be elevated in saliva of periodontitis patients [31]. Since sTLRs are thought to function as decoy receptors for their respective PAMPs, we measured sTLR-2 and sTLR-4 in saliva. We observed that while both sTLR-2 and sTLR-4 showed a trend toward significance between both cohorts, only the reduction in sTLR-4 was significant (P ≤0.05; Fig 1C and 1D).

### Correlation of sTLR-2 and sTLR-4 with clinical parameters

**Fig 2:**
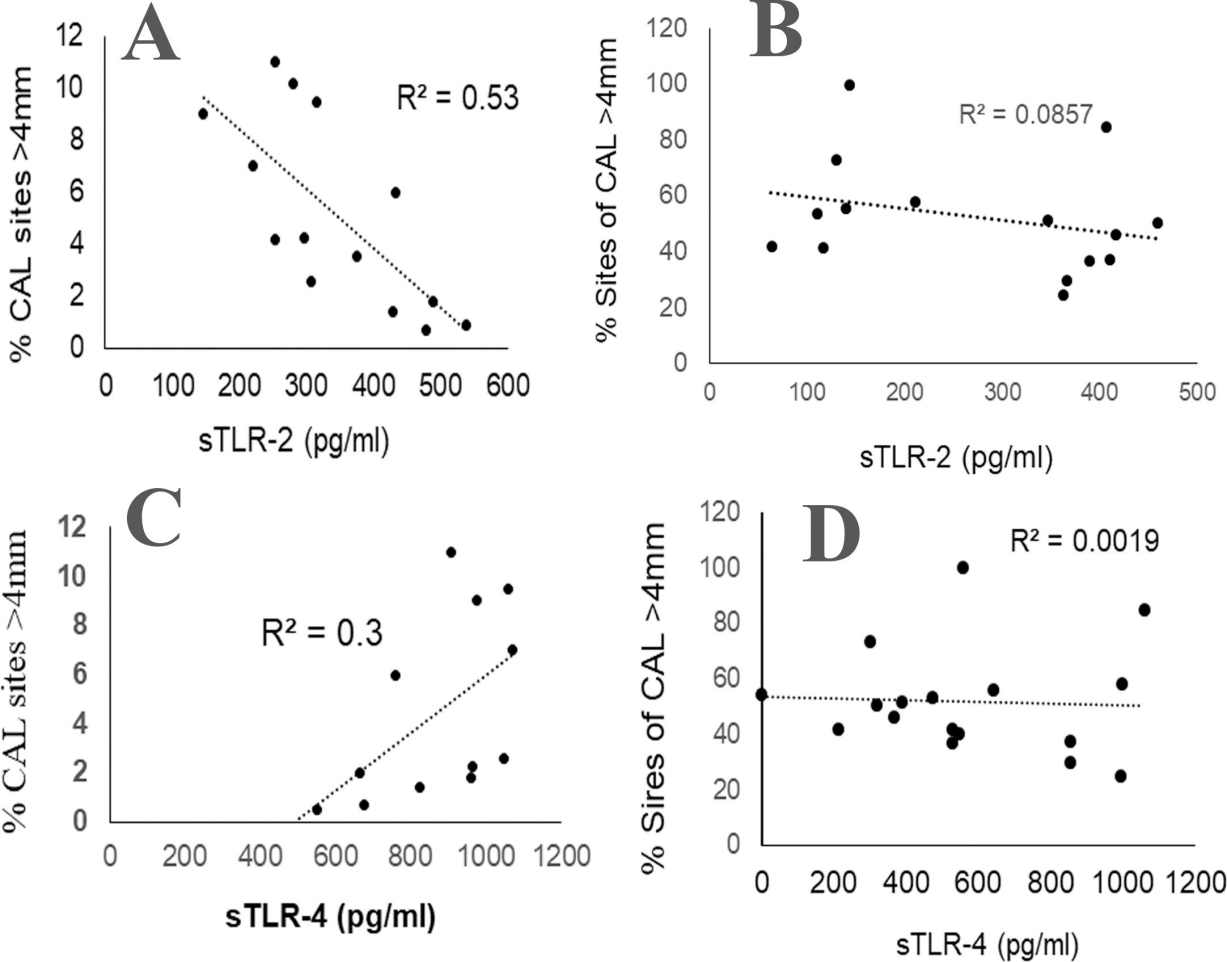
Relationship between the clinical periodontitis and sTLR-2 or sTLR-4 in clarified saliva. Unstimulated whole saliva (UWS) was collected from 20 patients with gingivitis (CAL 1-2mm not >15%, low % BOP sites) and 20 patients with chronic periodontitis (CAL >4m in >30% sites, high % BOP). (A) Pearson correlation between the % CAL and sTLR-2 (A,B) or sTLR-4 (C,D) in gingivitis (A,C) and chronic periodontitis (B,D).

To evaluate whether the salivary sTLR-2 or sTLR-4 could be potential biomarkers for CP, we estimated the correlation coefficient between the salivary sTLR-2 or sTLR-4 with the paired clinical parameters. Interestingly, an inverse correlation was observed between the salivary sTLR-2 and the paired clinical parameters of % CAL (r^2^ = 0.56) or % bleeding index (r^2^ = 0.63) in the gingivitis cohort (Fig 2A). In contrast, we observed a direct correlation between the salivary sTLR-4 and the % CAL sites (r^2^ = 0.3) and % bleeding index (r^2^ = 0.5) in the gingivitis cohort (Fig 2C). However, neither the sTLR-2 nor the sTLR-4 in saliva correlated with the paired % CAL or % bleeding index in the periodontitis cohort (Fig 2B and 2D). Taken together, these results suggest that sTLR-2 and sTLR-4 could potentially represent markers of gingivitis prior to bone loss.

### Epithelial cell expression of TLR-2 and TLR-4 transcripts

**Fig 3:**
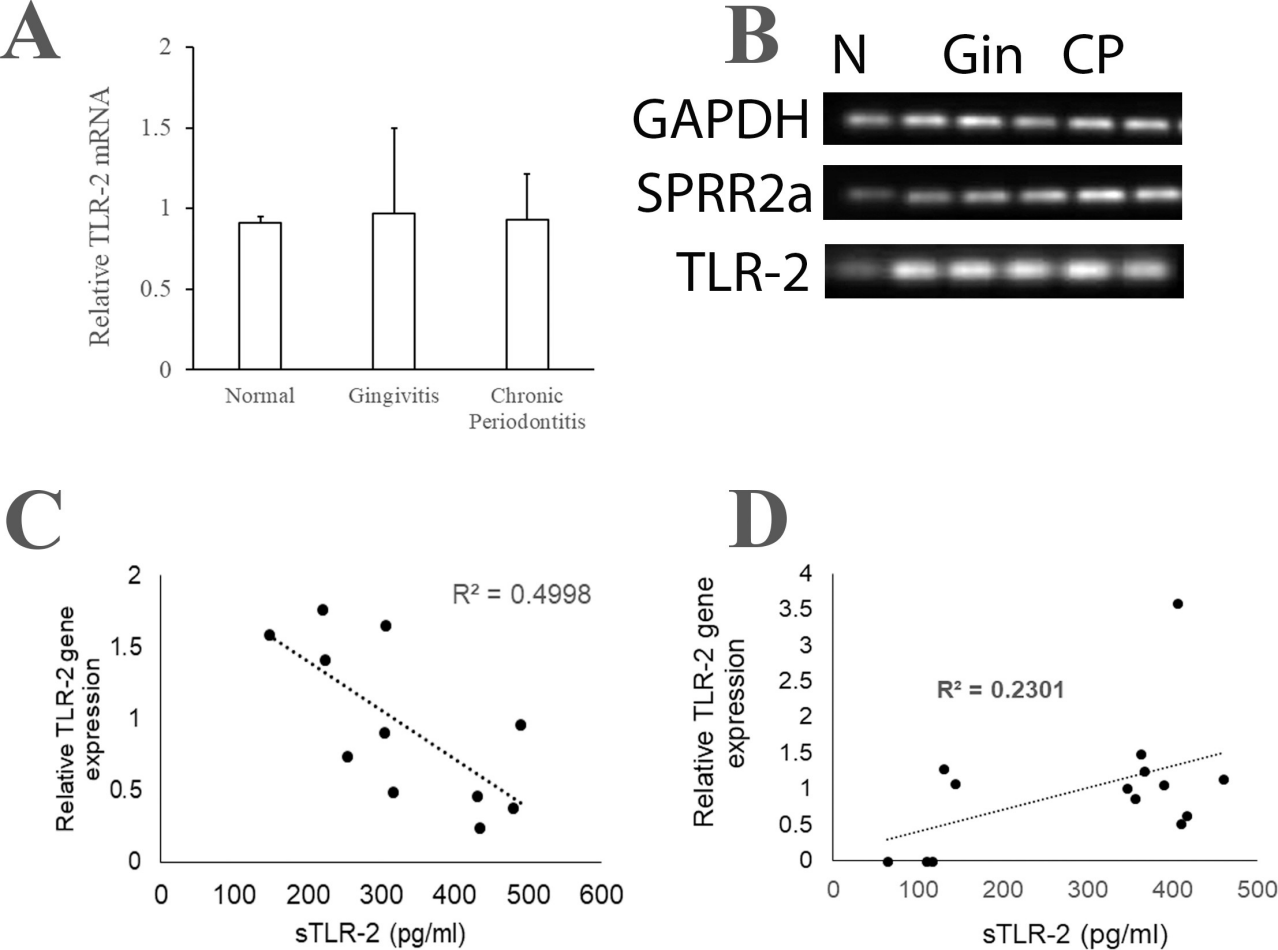
Relationship between the sTLR-2 and the paired SEC associated TLR-2 transcript. Unstimulated whole saliva (UWS) was collected from 10 individuals with normal oral health, 20 patients with gingivitis and 20 patients with chronic periodontitis. UWS samples were processed to separate clarified and epithelial cell rich fraction as described in materials and methods. The concentration of sTLR-2 was measured by ELISA. The SEC associated TLR-2 mRNA, the epithelial differentiation associated gene SPRR2a and the house keeping gene GAPDH was determined by quantitative PCR. Relative quantities of TLR-2 mRNA with respect to SPRR2a mRNA were determined using the 2^-ΔCt^ method. (A) Shows no significant difference in the TLR-2 mRNA between the three groups, (B) shows gel electrophoresis of the PCR products, (C) shown a moderate inverse correlation between sTLR-2 and SEC TLR-2 mRNA in the gingivitis cohort and (D) shows a mild direct correlation between the sTLR-2 and SEC TLR-2 mRNA in the periodontitis cohort. Cohorts are labelled as N: normal, Gin: gingivitis and CP: chronic periodontitis.

**Fig 4:**
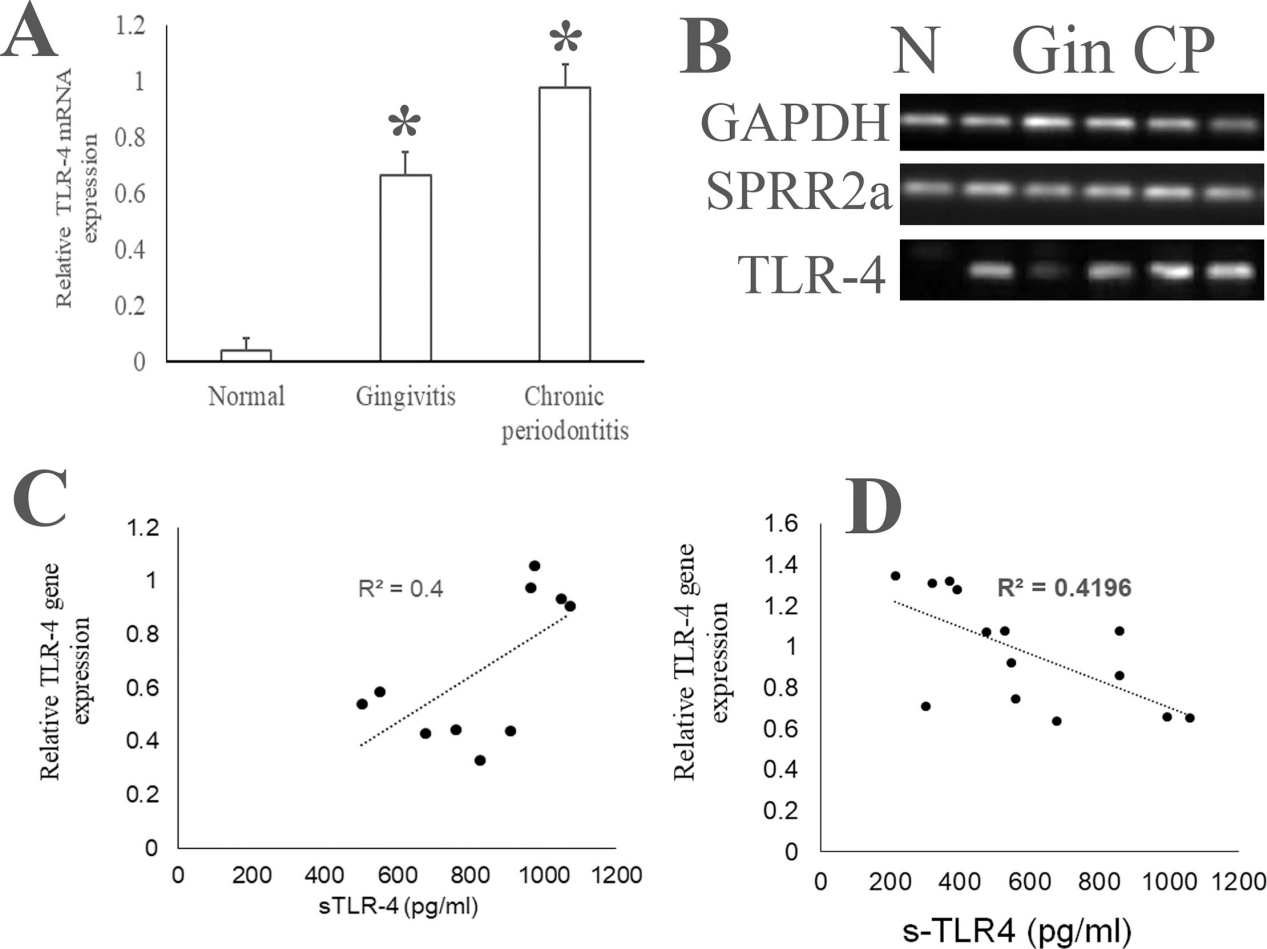
Relationship between the sTLR-4 and the SEC associated TLR-4 transcript. Unstimulated whole saliva (UWS) was collected from 10 individuals with normal oral health, 20 patients with gingivitis and 20 patients with chronic periodontitis (defined as the presence of 30% of sites >4 mm CAL). UWS samples were processed to separate clarified and epithelial cell rich fraction as described in materials and methods. The concentration of sTLR-4 was measured by ELISA. The SEC associated TLR-4, the epithelial differentiation associated SPRR2a and the GAPDH genes was determined by quantitative PCR. (A) Relative quantities of TLR-4 mRNA with respect to SPRR2a mRNA were determined using the 2^-ΔCt^ method. (A) Shows no significant difference in the TLR-4 mRNA between the three groups, (B) shows gel electrophoresis of the PCR products, (C) shown a moderate positive correlation between the sTLR-4 and the SEC TLR-4 mRNA in the gingivitis cohort and (D) shows a moderate inverse correlation between the sTLR-4 and the SEC TLR-4 mRNA in the periodontitis cohort. Cohorts are labelled as N: normal, Gin: gingivitis and CP: chronic periodontitis.

Previously, we observed that the relative expression of TLR-4 mRNA in SEC from periodontitis saliva is higher than that in SEC from healthy saliva [39]. In this study, we observed that the SEC from the normal, the gingivitis and the CP cohorts exhibit equivalent levels of TLR-2 mRNA (Fig 3A and 3B). On the other hand, the TLR-4 mRNA was significantly higher in the SEC from the UWS of the gingivitis and the CP cohort as compared to that in SEC from the healthy saliva samples (Fig 4A and 4B).

### Correlation of sTLR-2/sTLR-4 with SEC associated TLR-2/TLR-4

Since one of the mechanisms of sTLR generation is extracellular shedding of membrane associated TLR, we next investigated whether the sTLR-2 and the sTLR-4 levels correlated with the TLR-2 or TLR-4 transcripts in the associated SECs [46]. Evaluation of the relationship between the salivary sTLR-2 with the paired SEC associated TLR-2 mRNA in the gingivitis cohort suggest a moderate inverse correlation (r^2^ = 0.5) (Fig 3C). This observation provides additional evidence of the origin of sTLR-2 by shedding of the ectodomain of the membrane bound TLR-2. In contrast, there was a mild direct correlation between the sTLR-2 and the paired SEC associated TLR-2 mRNA (r^2^ = 0.3) in CP saliva (Fig 3D). In gingivitis saliva, lower concentrations of sTLR-4 correlated with lower TLR-4 mRNA levels in the paired SEC (r^2^ = 0.4) (Fig 4C). As opposed to this, in periodontitis saliva, the sTLR-4 concentration exhibited a moderate inverse correlation with the paired SEC associated TLR-4 mRNA level (r^2^ = 0.4) (Fig 4D).

## Discussion

The potential value of saliva as a biospecimen is gaining increasing significance with the extended applications in genomics, proteomics, epigenomics, microbiome and metabolomics [11, 24]. Similar to the use of colonocytes in stools for evaluating colitis [47, 48] and that of uroepithelial cells in urine as indicators of dysregulated glucose metabolism [49], molecular analyses of the epithelial cells in saliva could represent a viable strategy for monitoring the host responses in CP. In this study, we report the potential clinical value of the combined assessment of sTLR-2 and sTLR-4 together with the paired SEC associated TLR-2 and TLR-4 transcript as predictive indicators of CP.

Dysbiosis or imbalance in the relative abundance of the microbial species in the dental plaque biofilm leads to destructive inflammation in periodontitis [2, 50]. TLRs as primary microbe recognition receptors have been widely investigated in periodontitis [18, 19]. While the immunohistochemical or immunofluorescence studies have shown elevated TLR-2 and TLR-4 protein expression in the gingival epithelial cells in periodontitis [13, 21, 51], molecular analyses of TLR-2 and TLR-4 transcripts have yielded variable results [52–54]. The variabilities in the mRNA expressions are attributed to the extent of inflammation and evaluation of the gingival tissue consisting of epithelial cells as well as infiltrating leukocytes and activated fibroblasts that add to the TLR-2 and TLR-4 mRNA. Furthermore, variability in the composition of the dysbiotic biofilm could also contribute to the differential TLR profile in CP [55, 56]. While optimal TLR activation in response to a commensal biofilm is critical for healthy host-microbe homeostasis, dysregulated activation of TLRs dictated by the dysbiotic biofilm promotes development of chronic inflammation and tissue destruction [19, 57, 58]. In this context, evaluating the exfoliated epithelial cell associated TLR and the soluble isoform could represent a method to develop a personalized host-microbe profile in CP.

Soluble ectodomains have been shown to be generated for many TLRs, including TLR2, TLR4, TLR6 and TLR9, by several mechanisms such as alternative splicing and proteolytic cleavage [46, 59]. The sTLRs are thought to bind and attenuate PAMPs in the extracellular space preceding their engagement with specific pattern recognition receptors [17, 46]. Although both serum sTLR2 and sTLR4 are elevated in response to infection, the kinetics of increase is differentially regulated. Elevated plasma concentration of sTLR-4 has been shown to discriminate infectious from non-infectious inflammation correlating effectively with the C-reactive protein levels [60]. Lower plasma sTLR-2 has been reported in chronic diseases, such as asthma and colitis [61, 62]. Furthermore, in mice induced peritoneal inflammation the sTLR-2 has been shown to reduce bacteria-associated inflammation without abrogating microbial recognition [63].

Our data showed that the sTLR-2 in saliva exhibits a decreasing trend in CP. During innate immune responses, ectodomain shedding permits downregulation of responses triggered by pathogens or stressors [60]. Pertinently, it is interesting that our data demonstrates a negative correlation between the SEC associated TLR-2 mRNA and the sTLR-2 in the gingivitis cohort and not in the periodontitis cohort. This suggests that the sTLR2 and the SEC associated TLR-2 transcript may represent markers for periodontal health. Previously, it has been reported that in monocytes, microbial stimulation mediated negative correlation between the membrane-bound TLR-2 and the sTLR-2 [60, 63].

Although naturally occurring sTLR-4 has been suggested to arise as alternate splice variant, it is also possible that passive diffusion could contribute to the overall concentration of sTLR-4 in saliva [32, 64]. Previously, elevated sTLR-4 has been reported in periodontitis in plasma and saliva [32]. However, we observed reduced sTLR-4 in periodontitis saliva. The discrepancy could be attributed to differences in the nature of the sample (stimulated versus unstimulated saliva), disease status at the time of sample collection and differences in the sensitivity and specificity of the antibodies used. It has been suggested that the sTLR-4 bound in a complex with MD2, a protein associated with TLR-4 on the host cell surface, may block the interaction between membrane-bound TLR-4 and its ligands and thereby inhibit TLR-4 signaling [65]. The lower sTLR-4 in CP saliva combined with the higher TLR-4 mRNA in the associated SEC in our cohort could potentially reflect active sequestration of microbial ligands by sTLR-4 and the compromised ability to block epithelial TLR-4. Furthermore, the direct correlation between the higher sTLR-4 and greater SECs TLR4 mRNA in gingivitis saliva perhaps support the sentinel functions of TLR4.

In conclusion, host response associated with bacterial invasion directs the disease progression from gingivitis to CP [1]. Microbial adherence and invasion mediate increased exfoliation of epithelial cells supporting the presence of high concentration of epithelial cells in saliva of individuals with CP [36, 37]. Our data of moderate inverse correlation between the sTLR-2 and that of direct correlation of sTLR-4 with clinical parameters in CP suggests that the evaluation of both could function as clinically viable diagnostic markers. In addition, the reversal of correlation between the sTLR-2 and the sTLR-4 with the paired SEC in periodontitis as opposed to gingivitis suggest that the assessment of these measures will provide effective way to track disease progression and even response to therapy. Indeed, oral epithelial cells of the buccal mucosa have been shown as extracrevicular reservoirs of periodontal pathogens supporting the clinical value of SEC in CP [66, 67]. If supported in larger cohort, our approach will allow for evidence-based highly individualized diagnosis, and risk based treatments for periodontitis [11].
